# Chitosan-Based Composite Film Containing Cell-Free Supernatant of *Lactiplantibacillus plantarum* JC2211 for Chilled Pork Preservation

**DOI:** 10.3390/gels12090765

**Published:** 2026-08-26

**Authors:** Xiaoqing Sun, Dawen Qin, Perpetual Ogechi Onyeaka, Songsong Jiang, Jingguo Xu

**Affiliations:** 1 Key Laboratory of Tropical Translational Medicine of Ministry of Education, School of Public Health, Hainan Medical University, Haikou 571199, China; mx120231293@stu.yzu.edu.cn; 2 College of Tourism and Cuisine Science, Yangzhou University, Yangzhou 225127, China; 3 NHC Key Laboratory of Tropical Disease Control, School of Life Sciences and Medical Technology, Hainan Medical University, Haikou 571199, China

**Keywords:** Yangzhou pickles, active packaging, lactic acid bacteria, metabolites, degradable film, antibacterial, meat preservation

## Abstract

Traditional fermented vegetables are important resources for screening excellent antibacterial lactic acid bacteria (LAB). In this study, a strain of *Lactiplantibacillus plantarum* JC2211 with broad-spectrum antibacterial activity against *Salmonella* Typhimurium (*S.*T), *Staphylococcus aureus* (*S.a*), *Listeria monocytogenes* (*L.m*), and *Pseudomonas aeruginosa* (*P.a*) was isolated from Yangzhou pickles. The minimum inhibitory concentration (MIC) of its cell-free supernatant (CFS) against all four pathogens was 25 μL/mL. The strain exhibited favorable biosafety and strong environmental adaptability, tolerating 8% NaCl, pH 3.0–10.0, and 0.3% bile salt. The CFS exerted synergistic antibacterial effects via multiple pathways, including increasing cell membrane permeability, reducing membrane potential, decreasing intracellular ATP levels, and inducing ROS accumulation. Metabolite identification indicated that organic acids (dominated by D-lactic acid at 9.37%, citric acid at 8.00%, and phenyllactic acid at 4.42%) were the main components. Using chitosan (CS) as the base material, composite preservation films were fabricated by incorporating 10–50% CFS. The composite film containing 40% CFS (CS/CFS40) exhibited the optimal comprehensive performance, with ABTS and DPPH radical scavenging rates of 90.24% and 79.17%, respectively, along with satisfactory tensile strength (18.70 MPa) and elongation at break (22.93%). In chilled pork preservation, the CS/CFS40 film significantly inhibited the proliferation of spoilage microorganisms, and effectively retarded lipid oxidation. Moreover, the film suppressed the colonization of *L.m* and *S.*T on meat surfaces, with bacterial loads maintained at 2.07–2.67 lg CFU/g and 2.92–2.99 lg CFU/g, respectively, over 7 days of refrigeration. Collectively, the CS/CFS40 composite film extended the shelf life of chilled pork to approximately 6 days. This study provides a novel natural biological preservative and eco-friendly active packaging material for chilled meat preservation.

## 1. Introduction

Lactic acid bacteria (LAB) are the core functional microorganisms in traditional fermented foods. They produce a variety of metabolites, including organic acids, bacteriocins, hydrogen peroxide, and exopolysaccharides, which exhibit broad-spectrum inhibitory effects against multiple foodborne pathogenic bacteria. Accordingly, LAB are regarded as promising sources of natural food preservatives [1,2]. Among these metabolites, organic acids serve as the principal antibacterial components of the cell-free supernatant (CFS). In acidic environments, undissociated organic acids penetrate the bacterial cell membrane, disrupt the proton motive force, and interfere with intracellular energy metabolism. Due to its amphipathic structure, phenyllactic acid can directly interact with membrane phospholipids and membrane proteins, thereby damaging membrane integrity [3]. Bacteriocins synergize with organic acids to exert multi-targeted antibacterial effects, forming transmembrane pores or interfering with the synthesis of the cell wall precursor lipid II [4,5]. Compared with live bacterial preparations, CFS contains no viable cells and can prevent quality deterioration caused by secondary fermentation in food systems, showing superior applicability in food preservation [2,6].

Traditional fermented vegetables harbor stress-tolerant LAB populations owing to their natural high-salt and low-pH fermentation environments, making them important reservoirs for screening excellent antibacterial strains [7,8]. The LAB strains isolated from traditional pickle vegetables showed strong inhibitory activities against foodborne pathogens [9,10]. As a time-honored traditional fermented food in Jiangsu Province, Yangzhou pickles undergo multiple processes, including salting, dehydration, and sauce fermentation, forming a unique microbial ecological niche [11,12]. Several studies have preliminarily characterized the LAB community composition in Yangzhou pickles [13]; however, systematic screening of strains with broad-spectrum antibacterial activity and in-depth exploration of the underlying antibacterial mechanism of their CFS remains limited.

In the field of food packaging, bio-based degradable materials have emerged as important alternatives to petroleum-based packaging due to their environmental sustainability. Among these, chitosan (CS)—a natural cationic polysaccharide obtained from chitin deacetylation—stands out as an ideal film-forming matrix owing to its excellent biodegradability, biocompatibility, non-toxicity, and film-forming ability [14]. However, pure chitosan films often exhibit insufficient antibacterial efficacy and antioxidant properties for certain food preservation applications. To overcome these limitations, the incorporation of LAB-derived CFS into chitosan matrices has been proposed as a promising strategy to synergistically enhance antibacterial and antioxidant performance, while maintaining biodegradability and food-contact safety [15,16]. For instance, chitosan films incorporated with CFS of *Lacticaseibacillus paracasei* ALAC-4 exerted strong antifungal activity against molds and yeasts on cheese surface [16], and İncili et al. demonstrated that chitosan coating blended with *Pediococcus acidilactici* CFS effectively prolongs sausage shelf life [17]. These studies indicate that CS/CFS composite preservation films possess promising application potential for chilled meat preservation. However, research on the development of composite preservation systems using CFS from broad-spectrum antibacterial LAB as active ingredients is still relatively scarce.

The aim of this study was to isolate and characterize an antibacterial LAB strain from Yangzhou pickles, and evaluate the efficacy of CS-based composite films incorporating the LAB CFS as an active packaging material for chilled pork preservation. To achieve this, a *Lactiplantibacillus plantarum* strain with broad-spectrum antibacterial activity against *Salmonella* Typhimurium (*S.*T), *Staphylococcus aureus* (*S.a*), *Listeria monocytogenes* (*L.m*), and *Pseudomonas aeruginosa* (*P.a*) was isolated. The antibacterial action of its CFS was elucidated through analyses of membrane integrity, membrane potential, intracellular ATP content, and ROS levels. Furthermore, composite films were fabricated using CS incorporated with varying concentrations of CFS, and their physicochemical properties, antibacterial activity, and antioxidant capacity were evaluated. The optimized composite film was then applied in chilled pork preservation.

## 2. Results and Discussion

### 2.1. Inhibitory Effect of LAB Against Pathogenic Bacteria

**Figure 1:**
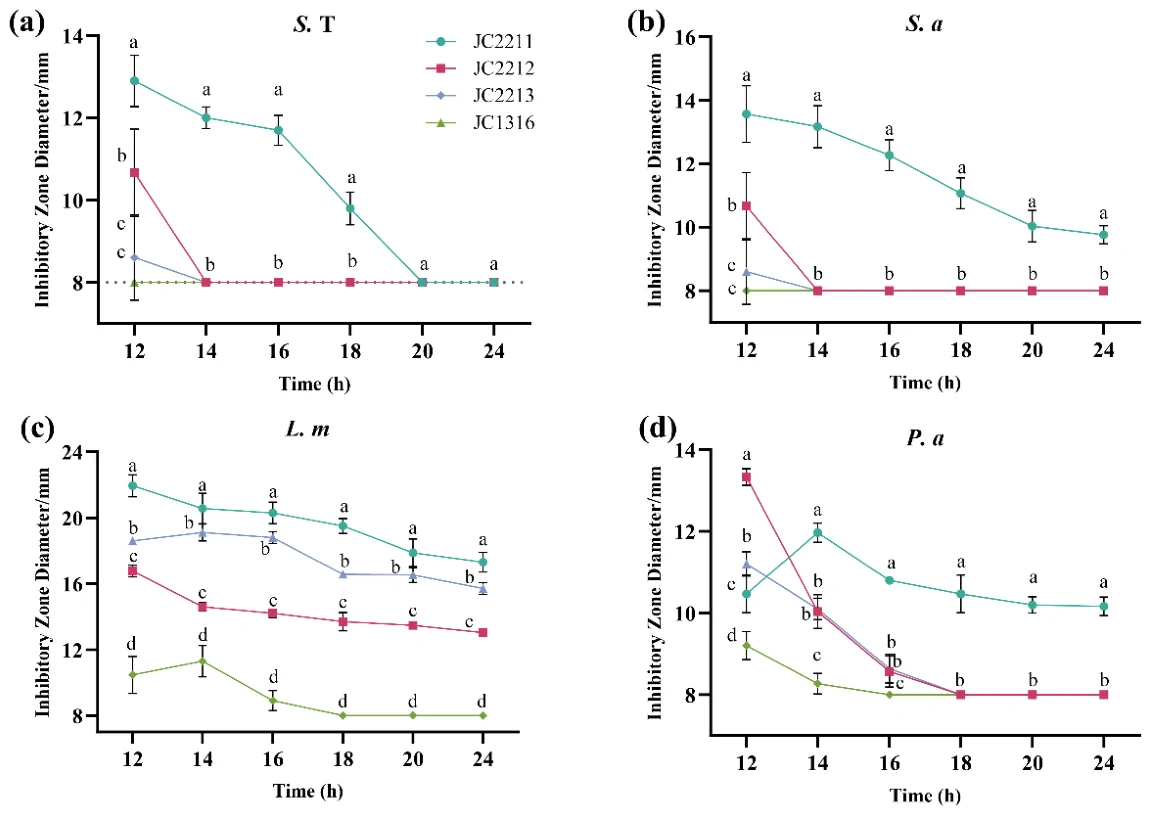
Inhibitory effects of cell-free supernatants from lactic acid bacteria isolated from Yangzhou pickles against (**a**) *Salmonella* Typhimurium (*S.*T), (**b**) *Staphylococcus aureus* (*S.a*), (**c**) *Listeria monocytogenes* (*L.m*), and (**d**) *Pseudomonas aeruginosa* (*P.a*). The dash line at 8 mm indicates the baseline for no antibacterial activity (outer diameter of the Oxford cup). Different lowercase letters denote significant differences in antibacterial activity among strains at the same time point (*p* < 0.05).

**Figure 2:**
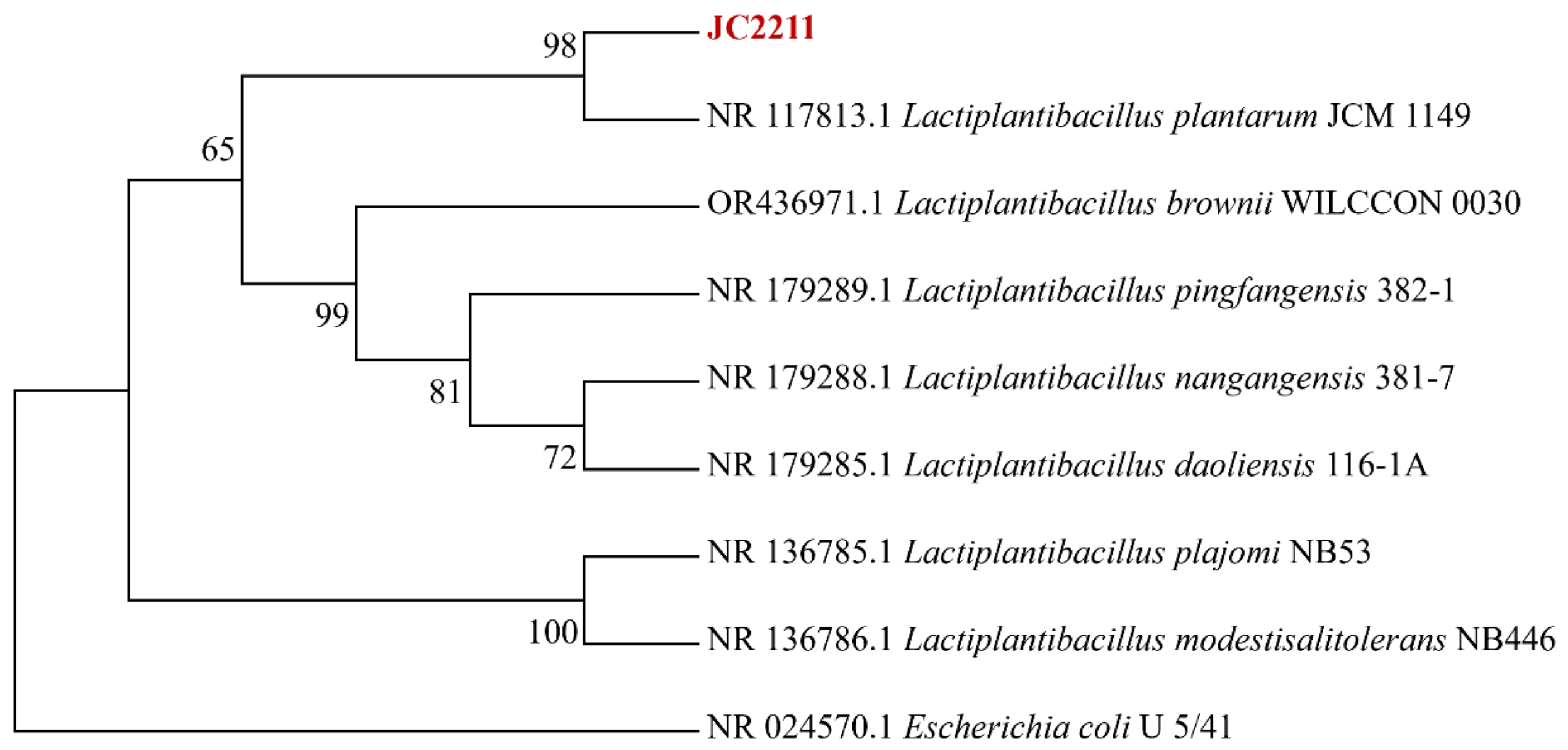
Phylogenetic tree of *Lactiplantibacillus plantarum* JC2211 based on 16S rRNA gene sequences.

A total of 25 lactic acid bacteria (LAB) strains were isolated from traditional Yangzhou pickles. Among them, 8, 16, 18, and 8 strains exhibited inhibitory effects against *S.T*, *S.a*, *L.m*, and *P.a*, respectively (Table S1). Four LAB strains, JC2211, JC2212, JC2213, and JC1316, displayed simultaneous inhibitory activity against all four pathogens. As shown in Figure 1, the antibacterial activity of CFS from the four LAB strains gradually declined over time. JC2211 presented the most prominent antibacterial capacity; at 12 h, the diameters of its inhibition zones against *S.*T, *S.a*, *L.m*, and *P.a* were approximately 12.90 mm, 13.57 mm, 21.93 mm, and 10.46 mm, respectively, and a relatively high antibacterial level was maintained until 24 h. The 16S rRNA gene sequencing results showed that JC2211 showed 99.93% sequence homology and a close genetic relationship with *Lactiplantibacillus plantarum* JCM1149. Accordingly, the strain JC2211 was designated as *L. plantarum* JC2211 (Figure 2).

### 2.2. Biosafety Assessment of L. plantarum JC2211

Hemolytic activity, proteolytic ability, coagulase activity, and antibiotic resistance of *L. plantarum* JC2211 were determined (Figure S1 & Table 1). The results showed that *L. plantarum* JC2211 exhibited γ-hemolysis with a negative hemolytic reaction, indicating that the strain lacked the ability to lyse erythrocytes. The proteolytic assay was negative, suggesting that the strain did not secrete extracellular proteases and had no potential risk of degrading host tissue proteins. Meanwhile, the coagulase test was negative, revealing that the strain could not produce coagulase to coagulate plasma fibrinogen, confirming its favorable biosafety. As the positive control, *S. aureus* showed positive results in hemolysis, proteolysis, and coagulase tests. These findings demonstrate that *L. plantarum* JC2211 satisfied the safety criteria for food-grade microorganisms in terms of hemolytic activity, proteolytic capacity, and coagulase activity, and could be used as a candidate functional strain for food preservation. In addition, antibiotic susceptibility was classified as susceptible (S), intermediate (I), and resistant (R) according to the CLSI M100 standards [18]. *L. plantarum* JC2211 was susceptible (S) to ampicillin and chloramphenicol, intermediate (I) to tetracycline, and resistant (R) to gentamicin and kanamycin (Table 1). The resistance phenotypes of this strain to gentamicin and kanamycin are consistent with the well-documented natural resistance characteristics of lactic acid bacteria against aminoglycoside antibiotics. These phenotypes are commonly attributed to the deficiency of cytochrome-mediated electron transport systems and alterations in cell membrane permeability [19,20]. However, it should be noted that the current study did not perform molecular screening for specific transferable resistance genes (e.g., *aph(3′)-IIIa*, *aac(6′)-Ie*, or *ant(6)-Ia*). Future studies will include molecular screening for these genetic determinants to further confirm the biosafety of this strain. Therefore, while the phenotypic data suggest a favorable safety profile, further genetic characterization is warranted to fully confirm the biosafety of *L. plantarum* JC2211 for food applications.

### 2.3. Biological Characteristics of L. plantarum JC2211

**Figure 3:**
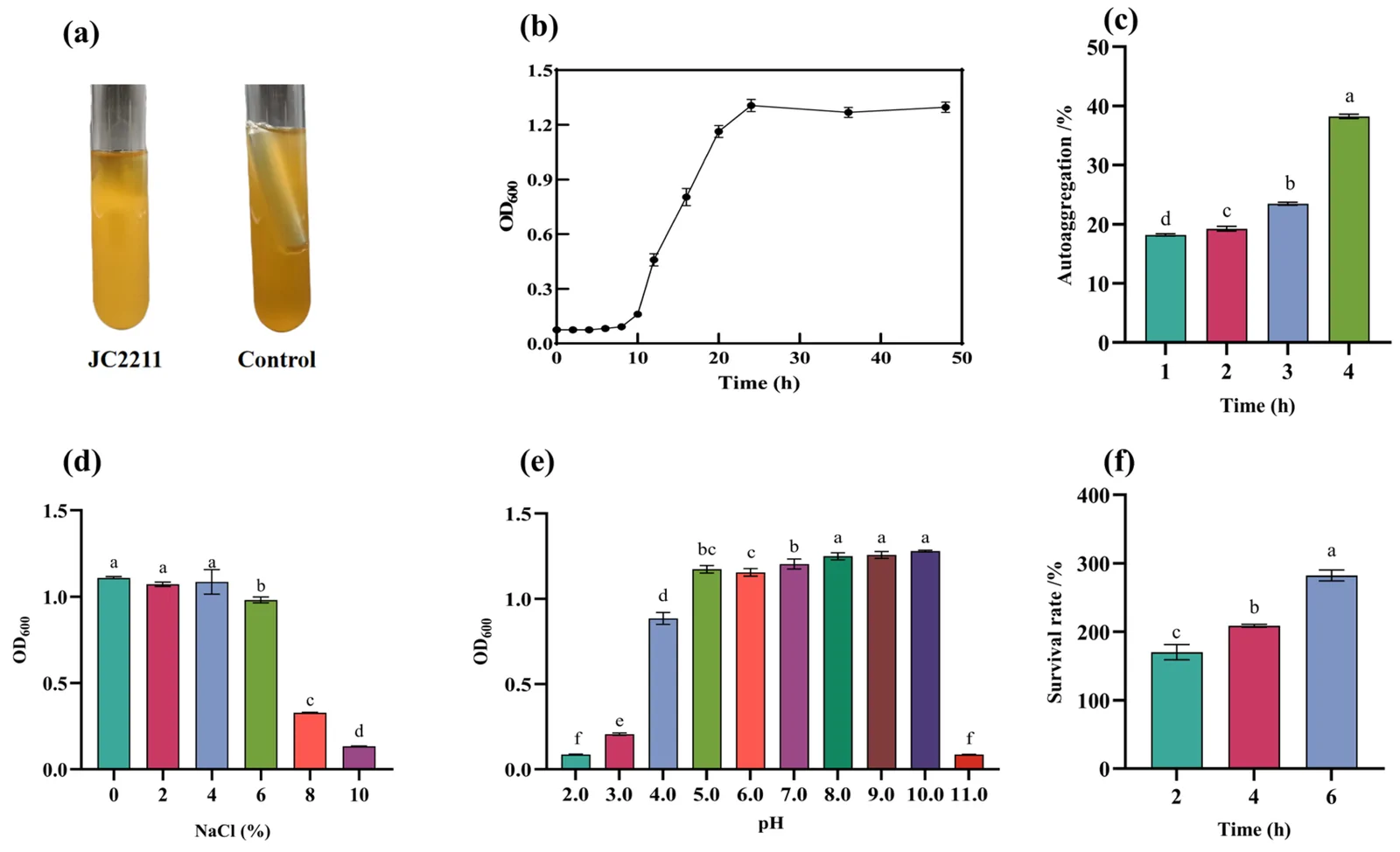
Physicochemical properties of *Lactiplantibacillus plantarum* JC2211: (**a**) gas production, (**b**) Growth curve, (**c**) autoaggregation ability, (**d**) growth at different NaCl concentrations, (**e**) growth at different pH values, and (**f**) survival rate at 0.3% bile salt. Different lowercase letters represent significant differences among groups (*p* < 0.05).

The gas production capacity of *L. plantarum* JC2211 is presented in Figure 3a. According to the results, no gas bubbles were observed in the Durham tube of *L. plantarum* JC2211. Subsequent metabolite identification revealed the presence of various organic acids, including D-lactic acid, citric acid, and phenyllactic acid (Table 2), confirming that *L. plantarum* JC2211 belongs to the facultative heterofermentative LAB group [21]. As shown in Figure 3b, the growth curve of *L. plantarum* JC2211 at 37 °C was divided into a lag phase (0–8 h), logarithmic phase (8–24 h), and stationary phase (24–36 h). Furthermore, Figure 3c showed that the autoaggregation rate increased gradually with the extension of incubation time, rising from 18.19 ± 0.19% at 1 h to 38.24 ± 0.37% at 4 h (*p* < 0.05). Figure 3d illustrates the growth of *L. plantarum* JC2211 in the presence of various NaCl concentrations, as indicated by the corresponding OD600 values. Overall, the OD600 value decreased as the NaCl concentration increased. At 0–4% NaCl, all OD_600nm_ values were greater than 1.0, with no significant differences between groups, suggesting that low salinity had no obvious inhibitory effect on the growth of the strain. Meanwhile, the OD600 decreased to approximately 0.9 with a slight reduction in biomass at 6% NaCl, though the strain continued to grow normally. As the NaCl concentration increased to 8%, the OD600 value decreased significantly (*p* < 0.05), although it remained higher than that of the 10% NaCl group. Furthermore, Figure 3e indicated that the strain grew well at pH 8.0–10.0 with OD_600_ values above 1.2. The OD_600_ decreased moderately at pH 5.0–7.0, remaining above 1.15. Growth was inhibited at pH 4.0, with an OD_600_ of around 0.88; at pH 3.0, the OD_600_ was only approximately 0.2. Almost no growth was detected at pH 2.0 and 11.0. In summary, the strain could grow within the pH range of 3.0–10.0, with optimal growth occurring at pH 8.0–10.0. Figure 3f showed that the survival rate of *L. plantarum* JC2211 increased from 175.38 ± 3.76% at 2 h to 287.56 ± 2.19% at 6 h in the presence of 0.3% bile salt, demonstrating that the strain could survive and proliferate under 0.3% bile salt stress. Therefore, *L. plantarum* JC2211 exhibited strong environmental adaptability, tolerating 8% NaCl, pH ranging from 3.0 to 10.0 and 0.3% bile salt. This was consistent with the reported tolerance characteristics of *L. plantarum* [22]. Its survival rate under bile salt stress was significantly higher than the initial level, possibly due to the synthesis of stress-protective proteins such as DnaK by the species [23].

**Figure 4:**
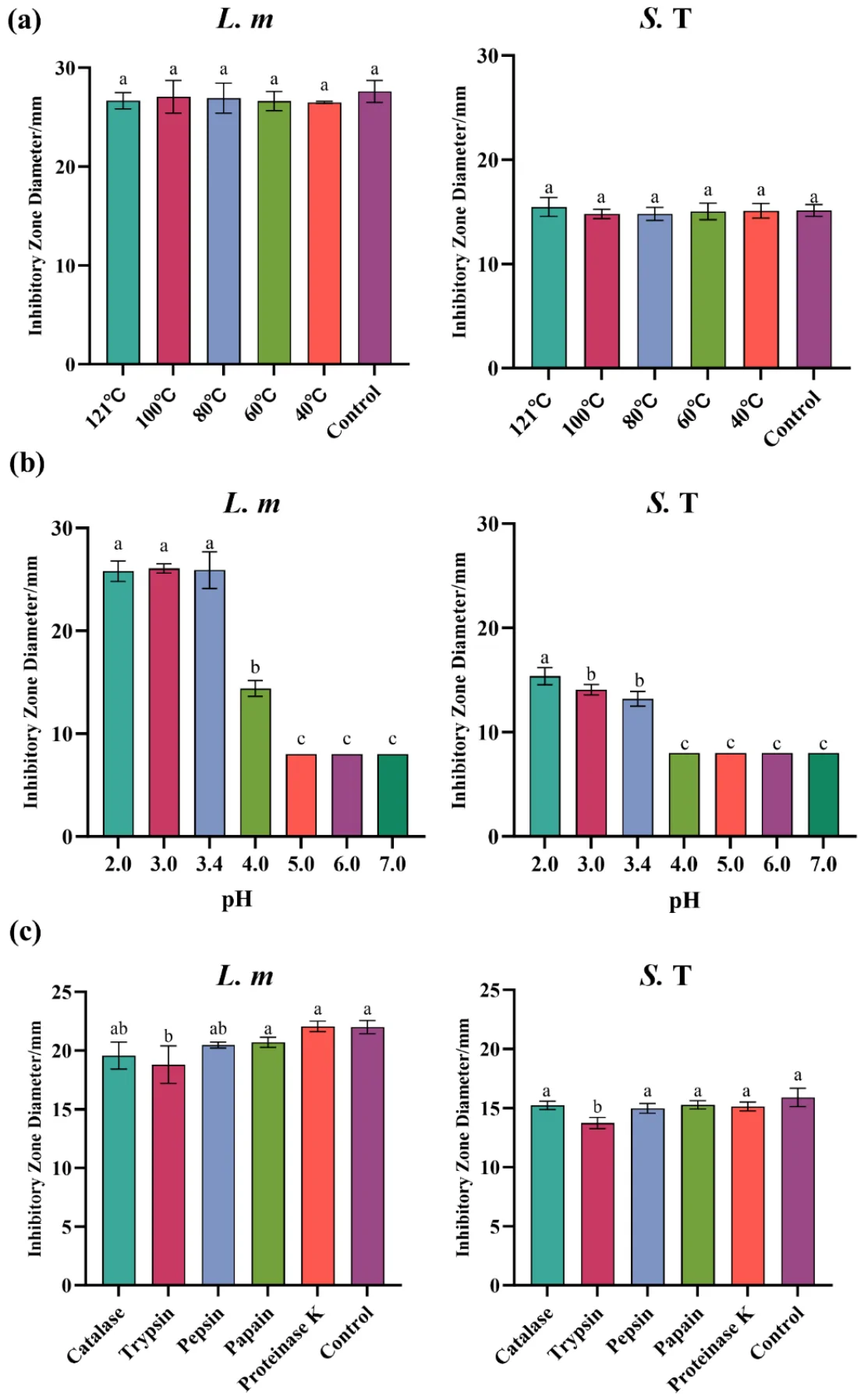
Effects of different treatments on the antibacterial activity of the cell-free supernatant (CFS) of *Lactiplantibacillus plantarum* JC2211 against *Listeria monocytogenes* (*L.m*) and *Salmonella* Typhimurium (*S.*T). (**a**) Thermal stability at different temperatures (40–121 °C); (**b**) pH stability at different pH values (2.0–7.0); (**c**) enzyme sensitivity following treatment with pepsin, papain, proteinase K, trypsin, and catalase. The outer diameter of the Oxford cup was 8 mm; an inhibition zone diameter of 8 mm indicated that CFS had no inhibitory effect on the tested pathogens. Different lowercase letters indicate significant differences in the inhibition zone diameters of the CFS against *L.m* and *S.*T (*p* < 0.05).

Metabolite profiling of the CFS of *L*. *plantarum* JC2211 revealed that 1623 metabolites were annotated via LC-MS/MS and categorized into 13 major classes (Figure S2). Carboxylic acids and their derivatives dominated the metabolite composition with a proportion of 30.27% (Table 2), which aligns with the typical metabolic trait of LAB that abundantly synthesize organic acids during fermentation. Organic acids presented the highest abundance, predominantly including D-lactic acid (9.37%), citric acid (8.00%), phenyllactic acid (4.42%), L-leucic acid (3.73%) and malic acid (0.12%). Moreover, multiple metabolites possessing potential antibacterial activity were identified, such as indolelactic acid (0.21%) and pentadecenoic acid (0.87%). Several amide compounds, namely oleamide, stearamide and lauramide, were also detected, with relative contents ranging from 0.11% to 0.24%. It should be noted that the present study did not perform targeted reconstitution experiments using organic acid standards at the exact concentrations identified in Table 2 to definitively ascertain whether these acids fully mimic the antibacterial capacity of the intact CFS. Nevertheless, as shown in Figure 4b, the near-complete loss of antibacterial activity upon pH neutralization provides strong evidence that organic acids are likely the primary antibacterial components. Recent studies have shown that in some *Lactiplantibacillus plantarum* strains, lactic acid alone is sufficient to mimic the antimicrobial activity of the CFS [24].

### 2.4. Influencing Factors of Antibacterial Activity of L. plantarum JC2211 CFS

To characterize the physicochemical stability of the antibacterial components in the CFS, *L.m* and *S.*T were selected as representative indicator strains. *L.m* was chosen as the most sensitive strain to the CFS, while *S.*T was selected as a representative Gram-negative enteric pathogen commonly associated with meat contamination. After treatment at 40 °C, 60 °C, 80 °C, 100 °C and 121 °C for 15 min, the antibacterial activity of the cell-free supernatant (CFS) of *L. plantarum* JC2211 against *L.m* and *S.*T showed no significant change (Figure 4a). These results suggest that the antibacterial components in the CFS are highly thermally stable and can maintain their antibacterial function over a broad temperature range, from room temperature to 121 °C. Huang et al. [25] and Santarelli et al. [26] reported that the antimicrobial components of the LAB are predominantly thermostable organic acids. The antibacterial activity of CFS was highly dependent on pH (Figure 4b). At pH 2.0–3.0, the inhibition zone diameter of CFS against *L.m* was approximately 25.9 mm, exhibiting the strongest antibacterial activity. However, the antibacterial activity gradually declined with an increase in pH. When the pH was adjusted to 4.0, CFS lost most of its inhibitory effect on *S.*T. A further increase in pH to 5.0 completely abolished the antibacterial activity against both *L.m* and *S.*T. These results suggest that the antibacterial substances in CFS are organic acids or acid-dependent active compounds. Enzyme stability assays revealed that the antibacterial activity of CFS did not change following treatment with pepsin, papain or proteinase K (Figure 4c). There was no significant difference between the catalase-treated group and the control group (*p* > 0.05), indicating that hydrogen peroxide is not the primary antibacterial component. Interestingly, trypsin treatment reduced the antibacterial activity of CFS against *L.m* and *S.*T by only 10.48% and 13.63%, respectively. This suggests that CFS may contain some proteins that have a synergistic antibacterial effect. It is noteworthy that the heat stability of the CFS (Figure 4a) does not preclude the presence of proteinaceous antibacterial components. Numerous bacteriocins have been reported to exhibit both high thermal stability (up to 121 °C) and sensitivity to proteolytic enzymes [27]. This combination of properties is attributed to the compact cyclic or highly ordered three-dimensional structures of many bacteriocins, which confer resistance to thermal denaturation, while proteolytic enzymes can still recognize and cleave specific peptide bonds within the active core [4]. Therefore, the partial loss of activity (~10–14%) upon trypsin treatment, together with the near-complete loss upon pH neutralization (Figure 4b), supports a model in which organic acids serve as the primary antibacterial components, while proteinaceous substances (likely bacteriocins) contribute synergistically in a secondary role.

### 2.5. Antibacterial Mechanism of L. plantarum JC2211 CFS

**Figure 5:**
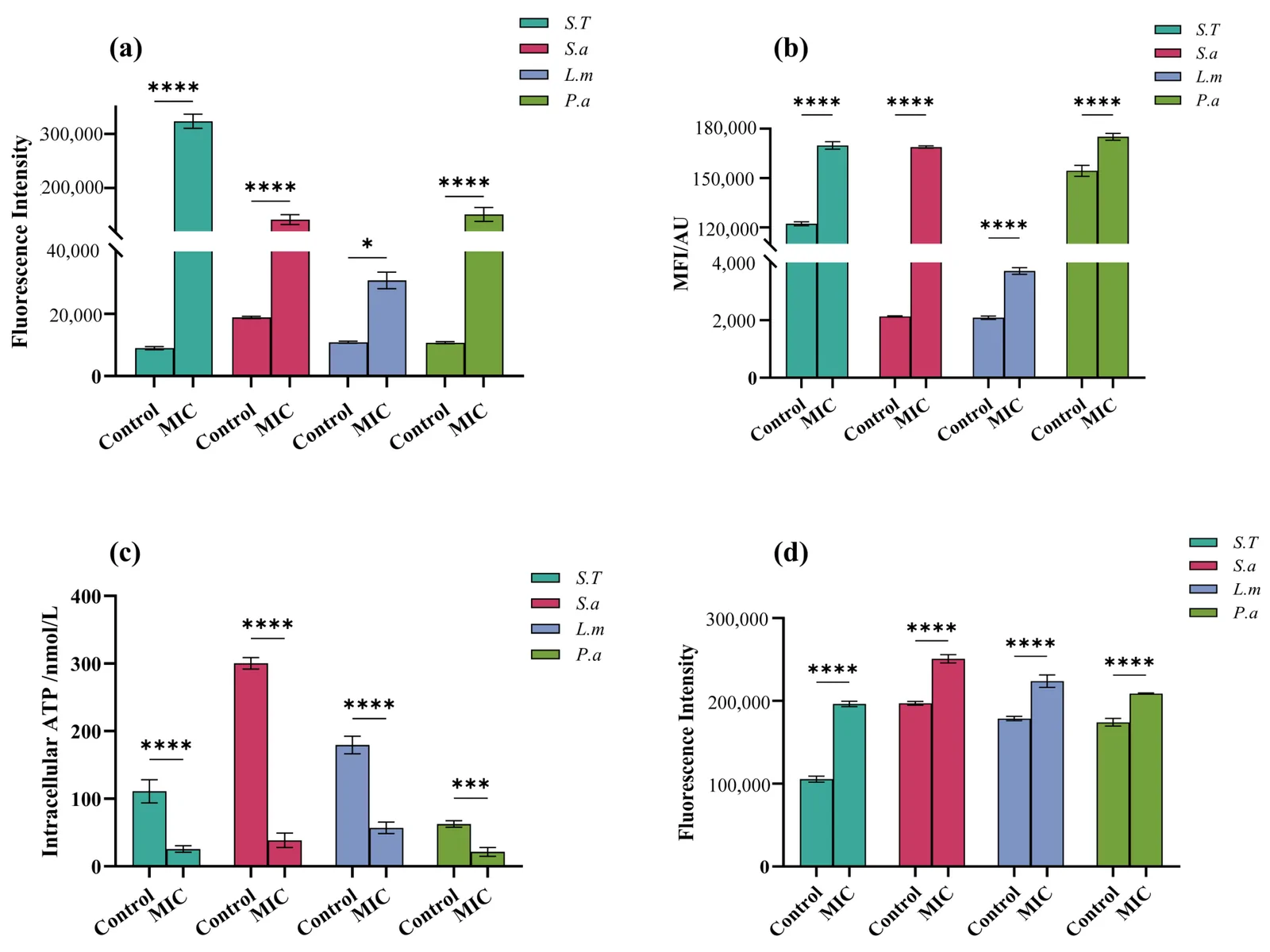
Effects of *Lactiplantibacillus plantarum* JC2211 cell-free supernatant (CFS) treatment at the minimum inhibitory concentration (MIC) on (**a**) cell membrane permeability, (**b**) membrane potential, (**c**) intracellular ATP content, and (**d**) intracellular ROS levels of four pathogenic strains. Asterisks indicate significant differences compared with the untreated control group for the corresponding strain: * *p* < 0.05, *** *p* < 0.001, **** *p* < 0.0001.

The minimum inhibitory concentration (MIC) of *L. plantarum* JC2211 CFS against four pathogens (*S.*T, *S.a*, *L.m*, and *P.a*) was determined to be 25 µL/mL. Following treatment with CFS at the MIC level, the propidium iodide (PI) fluorescence intensity of the four pathogenic strains increased significantly (*p* < 0.05) (Figure 5a). Compared to the control group, the fluorescence intensity of *S.*T, *P.a*, *S.a* and *L.m* increased by approximately 36.0-, 14.0-, 7.5- and 2.83-fold, respectively. This indicates that CFS can damage the cell membrane integrity of the four pathogens and increase their membrane permeability. In addition, DiSC_3_(5) fluorescence detection consistently revealed significant membrane depolarisation in four pathogens after CFS exposure (*p* < 0.0001) (Figure 5b). Among these, *S.a* exhibited the largest increase (approximately 7.93-fold), *P.a* the smallest (approximately 1.13-fold), and *S.*T and *L.m* increased by approximately 1.39- and 1.78-fold, respectively. This suggests that CFS markedly disrupted the membrane potential homeostasis of the tested pathogens. Furthermore, the intracellular ATP content of the four pathogens decreased remarkably after exposure to the CFS at the MIC level (*p* < 0.001), with the levels of ATP reduced by approximately 5.4-fold in *L.m*, 4.3-fold in *S.*T, 3.78-fold in *S.a* and 2.2-fold in *P.a* relative to the control group (Figure 5c). This demonstrates that CFS interferes with the intracellular energy metabolism of these pathogens. Additionally, the intracellular ROS fluorescence intensity of four pathogens was significantly increased following MIC CFS treatment (*p* < 0.0001), with increases of approximately 1.87-fold, 1.30-fold, 1.24-fold and 1.20-fold for *S.*T, *S.a*, *L.m* and *P.a*, respectively (Figure 5d). This indicates that CFS could induce oxidative stress and disrupt intracellular redox homeostasis, thereby disturbing the normal physiological metabolism of pathogenic cells. Metabolite analysis of *L. plantarum* JC2211 CFS identified multiple organic acids, including D-lactic acid, citric acid, and phenyllactic acid (Table 2). These compounds can penetrate bacterial cell membranes in their undissociated form and dissociate intracellularly to release protons, triggering intracellular acidification and inhibiting the activities of key glycolytic enzymes [28,29]. Meanwhile, these organic acids dissipate the proton motive force (PMF) and disrupt the proton gradient required for ATP synthesis, thereby causing comprehensive disturbances in energy metabolism [30], which is consistent with the reduced intracellular ATP content and elevated membrane permeability observed in this study. Moreover, indolelactic acid (ILA) detected in CFS is capable of inducing membrane potential depolarization and disrupting the electron transport chain [31], whereas indolepropionic acid (IPA) can trigger excessive ROS accumulation [32]. Long-chain fatty acid derivatives such as pentadecenoic acid and lauramide can insert into the bacterial lipid bilayer and impair membrane integrity [33,34,35,36], which further supports the membrane depolarization and massive ROS overproduction detected after CFS treatment.

### 2.6. Antibacterial and Antioxidant Abilities of CS/CFS

**Figure 6:**
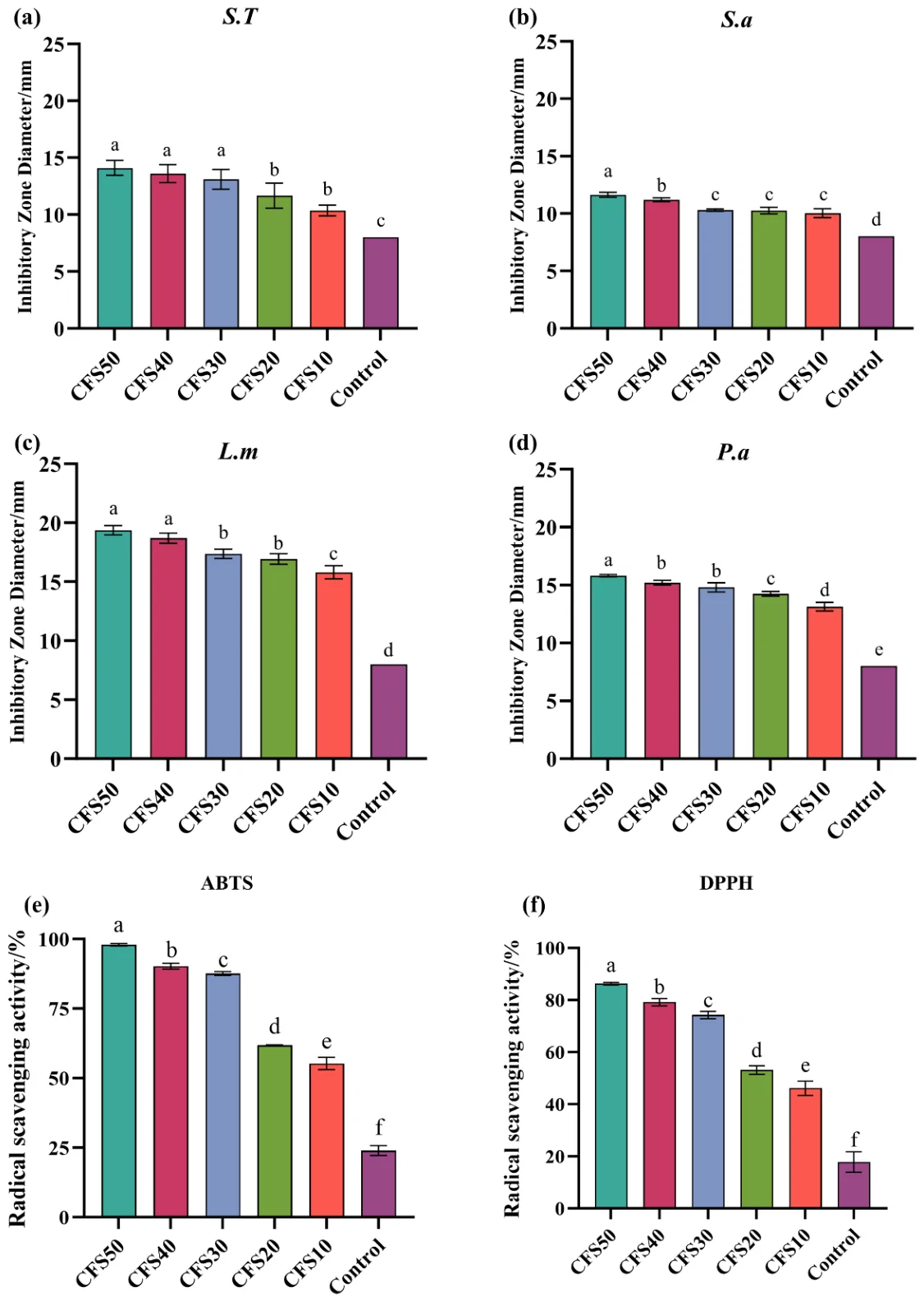
Antibacterial and antioxidant abilities of the chitosan composite film incorporated with different concentrations of *Lactiplantibacillus plantarum* JC2211 cell-free supernatant. (**a**–**d**) Inhibitory effects of CS/CFS against *Salmonella* Typhimurium (*S.*T), *Staphylococcus aureus* (*S.a*), *Listeria monocytogenes* (*L.m*), and *Pseudomonas aeruginosa* (*P.a*). (**e**,**f**) Antioxidant activities of CS/CFS evaluated by ABTS and DPPH free radical scavenging rates. An inhibition zone diameter equal to the 8 mm outer diameter of Oxford cups represents no antibacterial activity against tested pathogens. Different lowercase letters denote significant differences in antibacterial or antioxidant activities among the groups with different CFS concentrations (*p* < 0.05). Chitosan without CFS was used as the control group.

The antibacterial activity of CS/CFS gradually increased in a dose-dependent manner with elevated CFS supplementation (Figure 6a–d). The composites containing 50% CFS (CFS50) showed the strongest antibacterial performance, while the 10% CFS group (CFS10) also exhibited evident antibacterial activity. Among the four tested pathogens, the CS/CFS exerted the most potent inhibitory effect against *L.m*, retaining high antibacterial capacity even at low CFS addition levels. The antibacterial efficacy against *P.a* continuously improved as the CFS proportion increased, with a significant difference observed between the CFS10 and CFS20 groups. For *S.*T, no significant differences in antibacterial activity were detected among groups when the CFS dosage exceeded 30%. The inhibitory effect against *S.a* was relatively moderate overall; however, groups with high CFS contents possessed significantly stronger antibacterial ability than those with low CFS levels. In addition, the pure CS showed no antibacterial effect against the four pathogenic strains. Furthermore, the antioxidant ability of CS/CFS was evaluated (Figure 6e,f). The ABTS radical scavenging ability increased significantly in a dose-dependent manner with the elevation of CFS content (*p* < 0.05). The ABTS scavenging rate rose from more than 50.0% in the CFS10 and CFS20 groups to 90.24% in the CFS40 group and 97.97% in the CFS50 group. The DPPH scavenging rates were 46.14% and 53.15% for CFS10 and CFS20, respectively, and increased markedly to 74.19% in CFS30, finally reaching 86.28% in the CFS50 group. It should be noted that the pure chitosan (CS) film, which also contained residual acetic acid from the dissolution step, exhibited no measurable antibacterial activity against the four tested pathogens under the assay conditions. This confirms that the antibacterial activity observed in the CS/CFS composite films is primarily attributed to the incorporated CFS components. The bioactive components (e.g., organic acids) found in CFS can suppress bacterial growth by disrupting the integrity of the cell membrane and triggering intracellular acidification (Table 2 & Figure 5). The amino and hydroxyl groups abundant in chitosan act as electron and hydrogen atom donors, scavenging free radicals [37,38]. Meanwhile, organic acids derived from CFS further strengthen the antioxidant capacity via free radical scavenging and ferrous ion chelation [39]. Together, these complementary mechanisms of chitosan and CFS endow the CS/CFS composite film with excellent antibacterial and antioxidant performance.

### 2.7. Structural Characteristics of CS/CFS Composite Films

**Figure 7:**
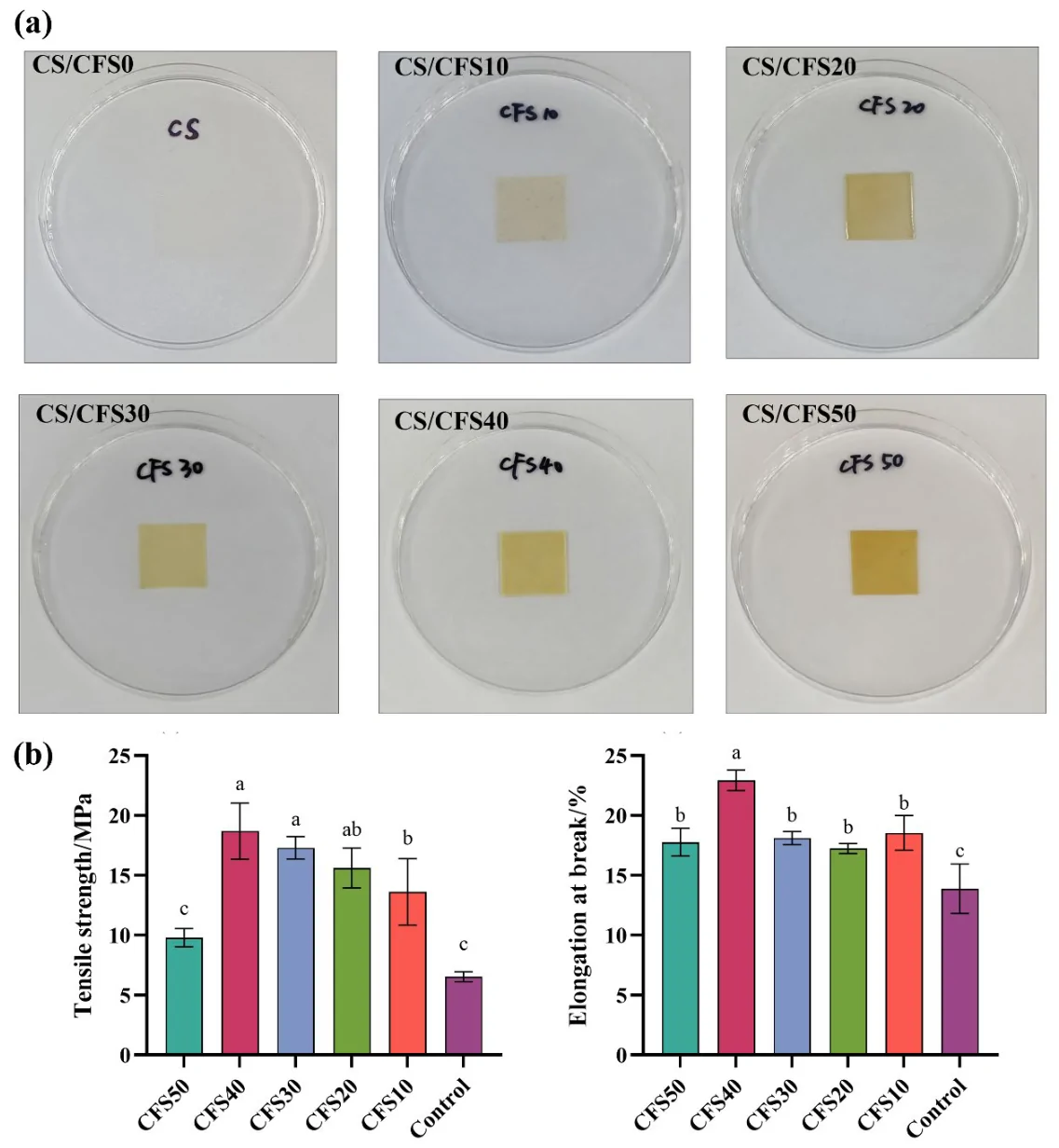
Macroscopic characteristics and mechanical properties of CS/CFS composite films. (**a**) Macroscopic appearance of composite films supplemented with different CFS concentrations; (**b**) Changes in tensile strength and elongation at break of the corresponding film. Different lowercase letters denote significant differences in antibacterial or antioxidant activities among the groups with different CFS concentrations (*p* < 0.05).

**Figure 8:**
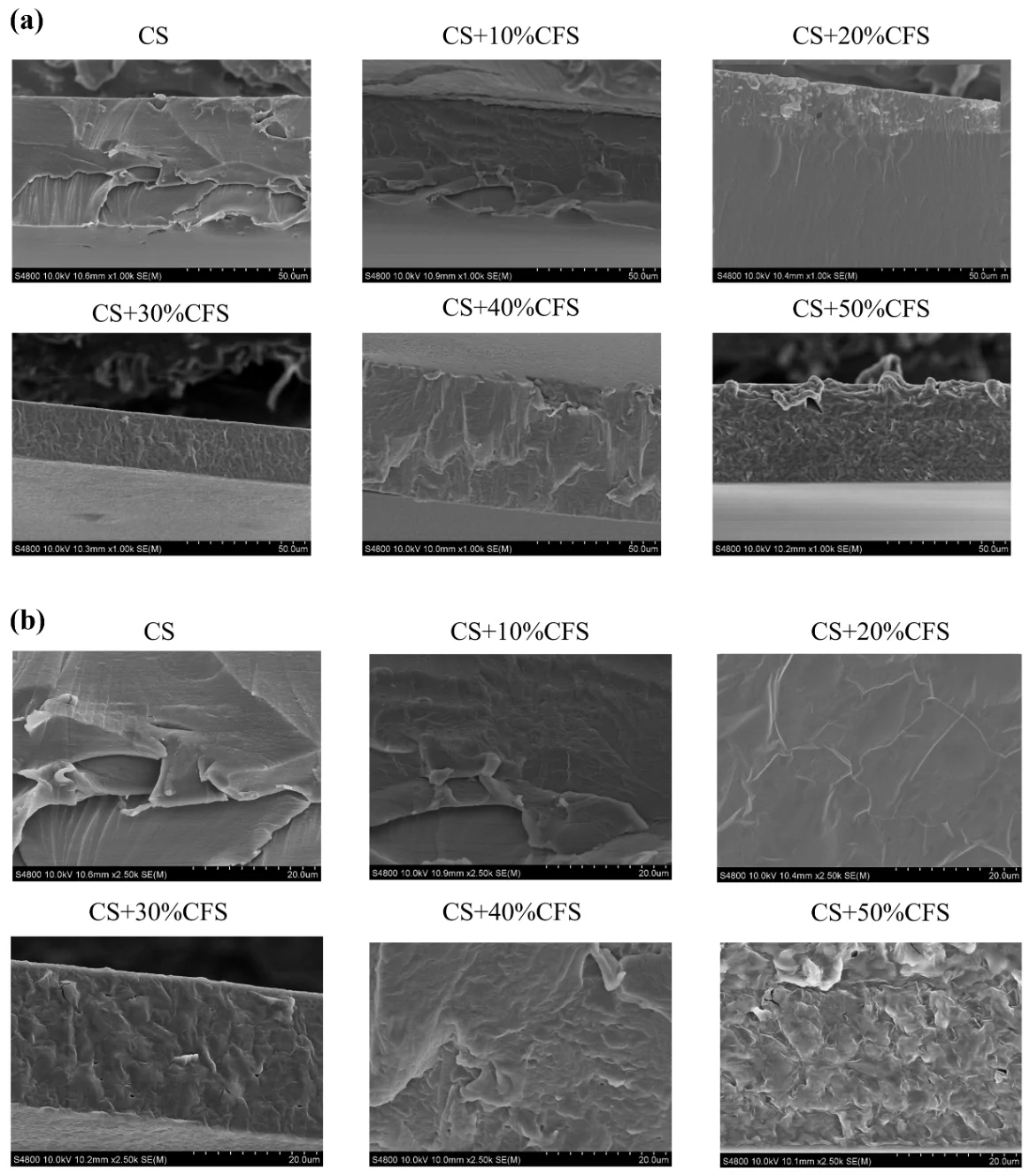
Cross-sectional microstructure of chitosan composite films incorporated with different CFS concentrations observed by SEM. (**a**) 1000× magnification; (**b**) 2500× magnification.

The results showed that all films had thickness values in the range of 72–106 μm (Table S2), and no statistically significant differences were observed among the control and CFS-containing groups (*p* > 0.05), confirming that CFS incorporation up to 50% did not significantly alter film thickness. The macroscopic morphological features of CS/CFS composite films are presented in Figure 7. The chitosan film without CFS exhibited a transparent appearance. With the incorporation and increasing content of CFS, the color gradually changed from transparent to orange-brown (Figure 7a). Tensile strength increased as the CFS proportion rose from 10% to 40%, while it decreased in the CFS50 group (Figure 7b). The control group showed the lowest elongation at break (13.89%), and no significant differences were observed among the CFS10 to CFS30 groups. The CFS40 group reached the maximum elongation at break of 22.93%, whereas the value decreased to 17.76% in the CFS50 group (Figure 7b). Furthermore, the cross-sectional microstructure of CS/CFS composite films were observed by scanning electron microscopy (SEM) (Figure 8). The chitosan film without CFS displayed a smooth, compact morphology. As the CFS content increased, the cross-sectional roughness increased. When the CFS content reached 50%, the film exhibited obvious structural heterogeneity and the dense microstructure was significantly disrupted. These microstructural changes were highly consistent with the variations in tensile strength and elongation at break. The decline in mechanical properties at 50% CFS can be attributed to two interrelated mechanisms: excessive plasticization by organic acids and disruption of the chitosan network structure. At lower CFS concentrations (10–40%), organic acids act as moderate plasticizers by intercalating between chitosan chains, enhancing chain mobility and elongation at break while maintaining adequate tensile strength [40]. However, the excessive organic acid concentration over-plasticizes the film, disrupting the hydrogen bonding network to the extent that the chitosan chains lose structural coherence [41]. Concurrently, the large volume fraction of CFS components exceeds the compatibility limit with the chitosan matrix, leading to aggregation and phase separation, as evidenced by the SEM cross-sections (Figure 8). Overall, the CS/CFS40 composite film demonstrated optimal characteristics in terms of antibacterial activity, antioxidant capacity, and mechanical performance among all tested formulations.

### 2.8. Inhibitory Effect of CS/CFS40 Composite Film on L. m and S. T in Pork

**Figure 9:**
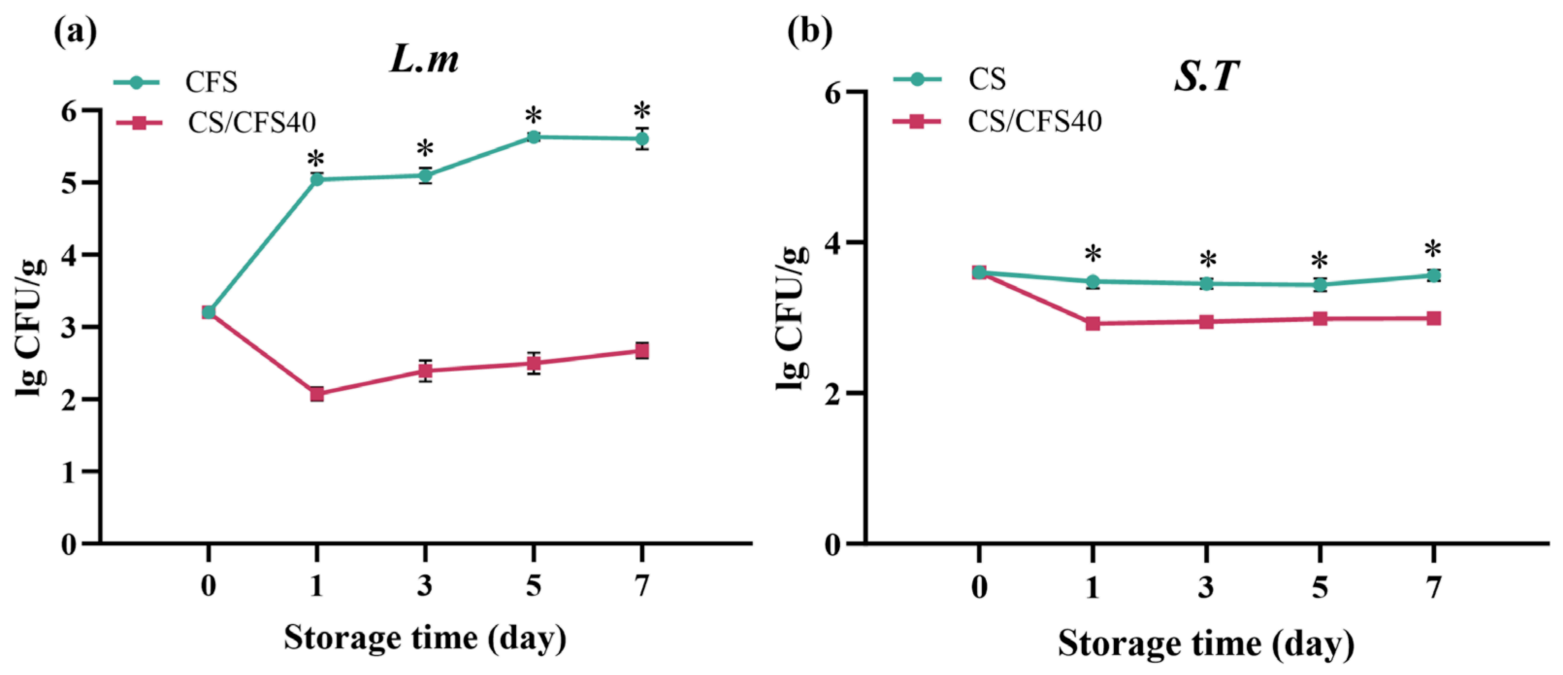
Inhibitory effects of chitosan composite film loaded with 40% cell-free supernatant (CFS40) on the proliferation of foodborne pathogens on chilled pork. (**a**) *Listeria monocytogenes* (*L.m*); (**b**) *Salmonella* Typhimurium (*S.T*). The chitosan (CS) film without CFS was used as the control group. Asterisks indicate significant differences between the CS/CFS40 and control groups at the same time point (*p* < 0.05).

The results of the simulated pathogenic contamination assay are presented in Figure 9. The CS/CFS40 composite film exhibited a remarkable inhibitory effect on the proliferation of *L.m* and *S.*T on pork surfaces. During 7 days of refrigerated storage, the viable count of *L.m* in the control group (CS film without CFS) increased from 5.1 lg CFU/g on day 3 to 5.60 lg CFU/g by day 7. In comparison, the CS/CFS40 group maintained a stable bacterial load ranging from 2.07 to 2.67 lg CFU/g. For *S.*T, the bacterial population in the control group increased to 3.56 lg CFU/g on day 7. By contrast, the CS/CFS40 group sustained a relatively stable count between 2.92 and 2.99 lg CFU/g. Significant differences were detected between the control and CS/CFS40 group throughout the storage period (*p* < 0.05).

### 2.9. Application of CS/CFS40 Composite Film in Chilled Pork Preservation

#### 2.9.1. pH, Total Viable Count, TVB-N, and TBARS

**Figure 10:**
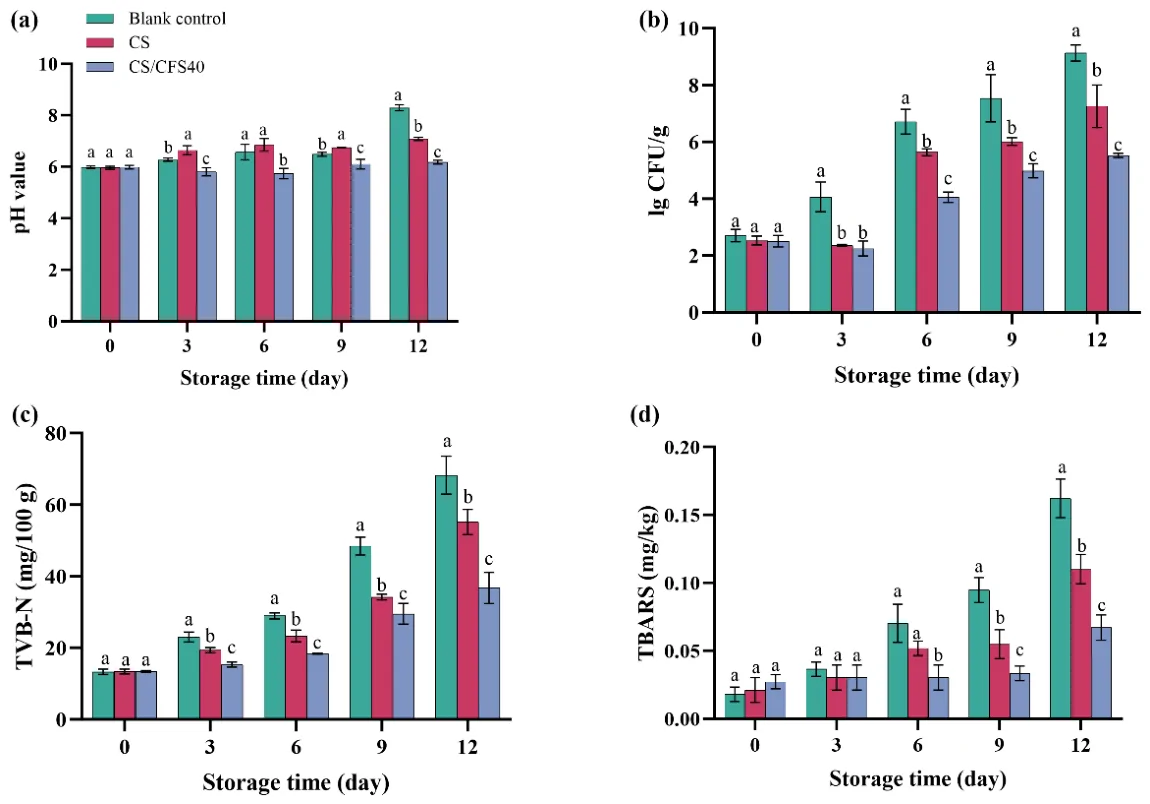
Changes in (**a**) pH value, (**b**) total viable count (TVC), (**c**) total volatile basic nitrogen (TVB-N), and (**d**) thiobarbituric acid reactive substances (TBARS) of pork during storage. Different lowercase letters indicate significant differences among groups at the same storage time (*p* < 0.05). The unwrapped pork served as the blank control. CS represents pure chitosan film, and CFS refers to chitosan film incorporated with 40% CFS.

The pH values of pork samples gradually increased during the 12-day storage period (Figure 10a). The CS/CFS40 group maintained the lowest pH from 3 d to 9 d, with significant differences compared with the blank control (unwrapped pork) and CS film groups. On day 12, the pH of the blank control exceeded 8.2, while the value for the CS/CFS40 group was only 6.18, which remained below the meat spoilage threshold of 6.4, confirming that the composite film could effectively retard pH elevation in chilled pork. In addition, the low pH environment generated by the film can change the chemical state of myoglobin and preserve the red color of meat [42,43]. The total viable count (TVC) continuously increased in all groups throughout storage (Figure 10b). On day 6, the TVC of the blank control reached 6.7 lg CFU/g. The CS/CFS40 group presented a lower TVC of 4.06 lg CFU/g, which was significantly different from the other two groups. On day 12, the TVC of the CS/CFS40 group was 5.52 lg CFU/g, demonstrating that the composite film suppressed the proliferation of spoilage microorganisms. The spoilage microbes were thriving in blank controls by day 12, which likely accounted for the rising pH above 8.2. These proteolytic bacteria break down meat proteins and amino acids through deamination to produce ammonia and alkaline nitrogen substances [44]. The total volatile basic nitrogen (TVB-N) contents are shown in Figure 10c. The blank control had a TVB-N value of 23.03 mg/100 g on day 3, while the CS and CS/CFS40 groups recorded 19.39 mg/100 g and 15.33 mg/100 g respectively. On day 6, the TVB-N values of the blank control and pure CS group increased to 28.93 mg/100 g and 23.33 mg/100 g, respectively, while the CS/CFS40 group remained at 18.34 mg/100 g. The thiobarbituric acid reactive substances (TBARS) values of pork were evaluated during storage (Figure 10d). The blank control had the fastest increase in TBARS, followed by the CS film, and the CS/CFS40 group consistently maintained the lowest TBARS level. Significant differences emerged between the CS/CFS40 group and the other two groups from day 6. On day 12, the TBARS value of the CS/CFS40 group was 58.58% lower than that of the blank control, indicating that the composite film effectively delayed lipid oxidation in chilled pork. The reduced TBARS accumulation in the CS/CFS40 group (Figure 10d) could be partially attributed to the physical oxygen barrier property of the dense chitosan film matrix, which synergistically acts with the chemical antioxidant activity of CFS to inhibit lipid oxidation [17]. Although direct oxygen permeability measurements were not conducted, the compact microstructure observed in SEM (Figure 8) and the sustained preservation efficacy during 12 days of refrigerated storage collectively suggest that the CS/CFS40 composite film possesses adequate barrier functionality for chilled pork applications. Future studies measuring oxygen transmission rate (OTR) would be beneficial to directly quantify this barrier property.

#### 2.9.2. Appearance and Texture Characteristics

**Figure 11:**
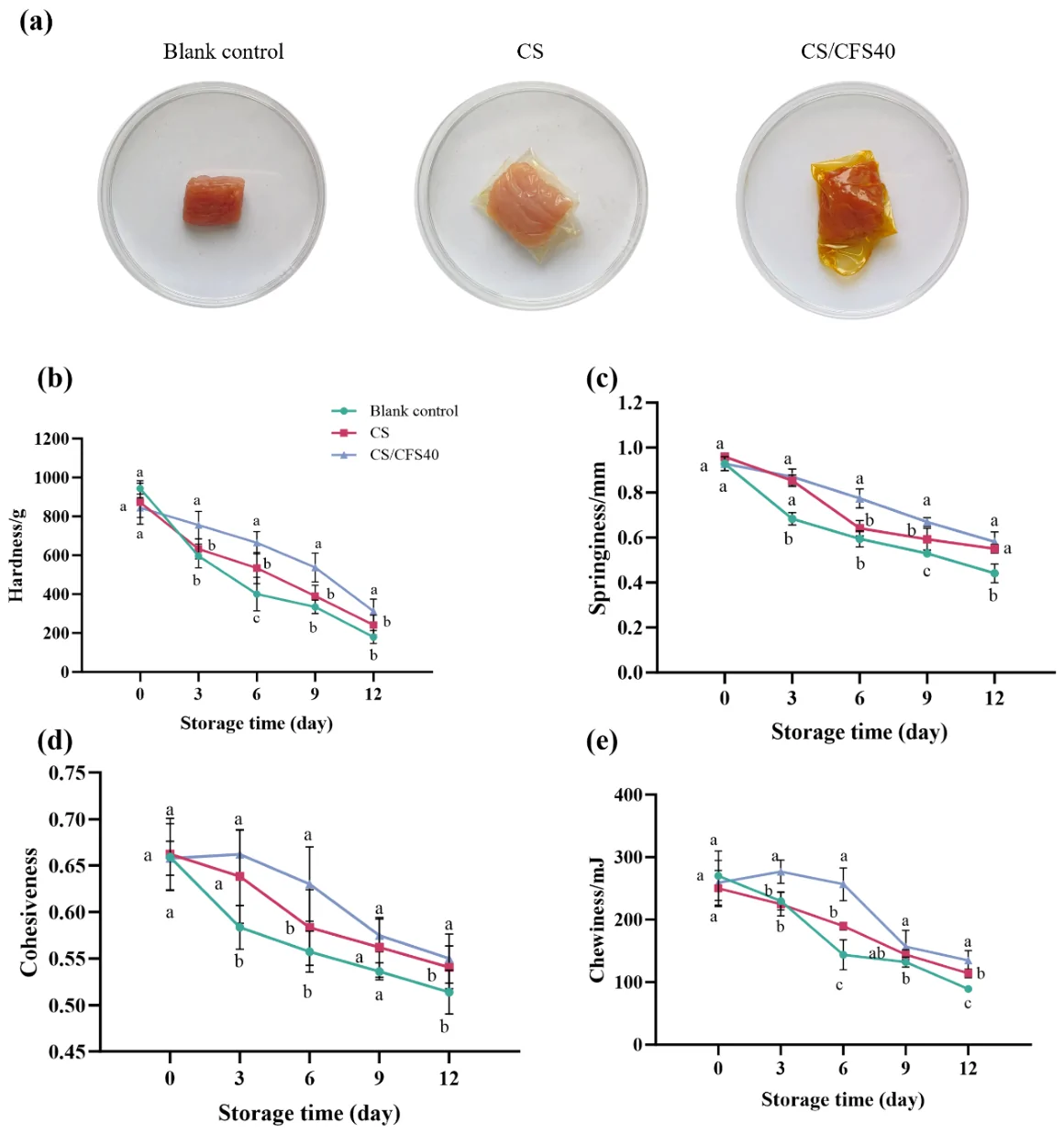
Appearance and texture characteristics of pork samples. (**a**) Initial appearance and changes in (**b**) hardness, (**c**) springiness, (**d**) cohesiveness, and (**e**) chewiness of pork during storage. Different lowercase letters indicate significant differences among groups at the same storage time (*p* < 0.05). The unwrapped pork served as the blank control. CS represents pure chitosan film, and CS/CFS40 refers to chitosan film incorporated with 40% CFS.

The initial appearance of pork samples from the blank control, pure CS and CS/CFS40 groups is presented in Figure 11a. The pure CS film possessed higher transparency than the CS/CFS40 composite film. Color of pork samples in all groups underwent distinct changes during storage (Table 3). The L* value (lightness) generally increased initially and then declined. From day 3 to day 9, the CS/CFS40 group displayed the stable L* levels, and no significant difference was detected between the blank control and pure CS groups, revealing that the composite film effectively stabilized pork lightness. The a* value (redness) decreased progressively over storage time, with the blank control showing the most remarkable reduction and the CS/CFS40 group the least. Significant differences in a* values were observed between the CS/CFS40 group and the other two groups during storage. This result indicated that the composite film likely retarded myoglobin oxidation and preserved the red color of pork [45]. As for the b* value (yellowness), the CS/CFS40 group exhibited minimal variation, and significant differences were confirmed between the CS/CFS40 group and the remaining two groups. These findings proved that the composite film effectively inhibited pork yellowing during storage. Collectively, the CS/CFS40 group achieved superior color stability of pork in terms of all three color indices compared with the pure CS and blank control groups.

Regarding textural properties of chilled pork, the declines in hardness, springiness, cohesiveness and chewiness proceeded more slowly in the CS/CFS40 group than in the blank control and pure CS groups over the entire storage period. In terms of hardness, the values of the blank control, pure CS and CS/CFS40 groups decreased by 5.24-, 3.74- and 2.61-fold on day 12 relative to the initial level, respectively. The CS/CFS40 group showed the mildest reduction in hardness, and no significant difference was detected between the pure CS and blank control groups (Figure 11b). For springiness, no significant difference was observed between the pure CS and CS/CFS40 groups on day 3, while a declining trend was already evident in the blank control group. On day 12, the CS/CFS40 group retained the optimal springiness, which remained at 39.01% of the initial value (Figure 11c). With respect to cohesiveness, significant differences were found between the CS/CFS40 group and the blank control group on days 3, 6 and 12. On day 12, cohesiveness of the three groups decreased by 22.03%, 18.28% and 16.39%, respectively, compared with their initial values (Figure 11d). For chewiness, the CS/CFS40 group maintained the highest values throughout storage, with significant differences from the other two groups at all sampling time points (Figure 11e). These findings demonstrate that the CS/CFS40 composite film effectively alleviates textural deterioration of chilled pork during storage. Furthermore, the favorable textural performance of pork can be attributed to the combined antibacterial and antioxidant activities of the composite film, which retards the degradation of muscle proteins [46,47]. Therefore, the CS/CFS40 composite film extended the shelf life of chilled pork to approximately 6 days and maintained key quality attributes (pH, TVC, and TBARS) below spoilage thresholds through day 9 of refrigerated storage. İncili et al. [17] incorporated chitosan with CFS from *Pediococcus acidilactici*, extending the shelf life of Frankfurt sausages to 28 days. Namazi et al. [48] used CFS from *Lactobacillus helveticus* as a coating film to effectively maintain the quality of refrigerated beef. These studies confirmed that combining CFS from LAB with bio-based materials can effectively preserve meat products.

## 3. Conclusions

In this study, *Lactiplantibacillus plantarum* JC2211 with broad-spectrum antibacterial activity was isolated and identified from Yangzhou pickles. Its cell-free supernatant (CFS) exerts potent antibacterial effects through multiple synergistic mechanisms, including disruption of cell membrane integrity, depolarization of membrane potential, depletion of intracellular ATP, and induction of oxidative stress. Physicochemical stability analyses and metabolite profiling demonstrated that organic acids likely act as the predominant antibacterial components, with proteinaceous substances playing a synergistic auxiliary role. A chitosan (CS)-based composite film incorporating 40% CFS was developed and demonstrated optimal performance in terms of antibacterial activity, antioxidant capacity, and mechanical properties (Figure 7). When applied to chilled pork preservation, the CS/CFS40 composite film effectively inhibited the growth of spoilage microorganisms and foodborne pathogens, retarded lipid oxidation, and extended the shelf life of the product to approximately 6 days (Figure 10 and Figure 11). This work not only provides a promising candidate strain and mechanistic insights into its antibacterial action but also offers a practical and biodegradable active packaging strategy for chilled meat preservation.

## 4. Materials and Methods

### 4.1. Materials and Reagents

Pickled vegetable samples, including mixed vegetables, preserved cucumber, Chinese artichoke and young ginger, were purchased from local markets in Yangzhou, Jiangsu province, China. *Salmonella* Typhimurium ATCC 13311, *Staphylococcus aureus* ATCC 6538, *Listeria monocytogenes* ATCC 19111, and *Pseudomonas aeruginosa* CMCC(B)10104 were preserved in the laboratory. All culture media were purchased from Qingdao Hope Bio-Technology Co., Ltd. (Qingdao, China) Analytical-grade chemicals were purchased from Sinopharm Chemical Reagent Co., Ltd. (Shanghai, China).

### 4.2. Isolation, Screening, and Identification of LAB

Briefly, 3 g of each sample was mixed with 27 mL sterile water. The mixtures were shaken at 120 r/min and 37 °C for 1 h, followed by serial dilution. Diluents were spread onto MRS agar containing 2% CaCO_3_, and incubated anaerobically at 37 °C for 48 h [49]. Single colonies with clear calcium-dissolving halos were picked and purified, and then stored in glycerol at −80 °C.

LAB isolates were primarily screened for antibacterial activity using the Oxford cup agar diffusion assay [50,51]. *S.*T, *S.a*, *L.m* and *P.a* were used as indicator strains. The bacterial suspension of each indicator strain was adjusted to an OD_600nm_ of 0.50 and spread on LB agar plates. A volume of 150 μL LAB cell-free supernatant (CFS) was added into the holes, and the plates were incubated at 37 °C for 24 h. All treatments were performed in three biological replicates. Isolates showing antibacterial activity with obvious inhibition zones were selected.

Genomic DNA was extracted using the TIANamp Bacteria DNA Kit (Tiangen Biotech co., Ltd., Beijing, China). The 16S rRNA gene was amplified by PCR using the primer set 27F/1492R. DNA sequencing was conducted by General Biotechnology Co., Ltd. (Chuzhou, China). Raw paired-end reads were assembled to generate the full-length 16S rRNA sequence. The resulting sequence was deposited in the NCBI database for species identification.

### 4.3. Safety Evaluation

The hemolytic activity was assessed on brain heart infusion agar supplemented with 5% defibrinated sheep blood (Beijing Solarbio Science & Technology Co., Ltd., Beijing, China). Plates were then incubated at 37 °C for 48–72 h, with *S. aureus* serving as the positive control [52]. Proteolytic activity was determined using PCA agar containing 1% skimmed milk powder, with *S.a.* used as the positive control [53]. For the coagulase test, the standardised bacterial suspension was mixed with 0.3 mL of lyophilised rabbit plasma (Beijing Solarbio Science & Technology Co., Ltd., Beijing, China) and incubated statically at 30 °C for 6 h [54]. Antibiotic susceptibility was tested via the Kirby–Bauer (K-B) disk diffusion method in accordance with CLSI M100 guidelines [18]. The tested antibiotics (Beijing Solarbio Science & Technology Co., Ltd., Beijing, China) included ampicillin (10 μg), chloramphenicol (30 μg), tetracycline (30 μg), gentamicin (10 μg), and kanamycin (30 μg). Following anaerobic incubation at 37 °C for 24 h, the diameters of the inhibition zones were measured.

### 4.4. Physiological Characteristics of L. plantarum JC2211

Gas production was determined using the Durham tube method [55]. The strain was cultivated anaerobically in 5% glucose-supplemented MRS medium at 37 °C for 24 h, after which gas production was observed visually. For the measurement of autoaggregation ability, the bacterial suspension was adjusted to an OD_600nm_ of 0.50 and then incubated statically at 37 °C. The absorbance of the supernatant was measured at one-hour intervals for 4 h. The autoaggregation rate was calculated in Formula (1) [56]. Among them, OD_t_ is the optical density of the supernatant at tested times; OD_0_ is the optical density at the initial time.

(1) $$\mathrm{Autoaggregation}\;\mathrm{rate}\;(\%)=\left(1-\frac{{\mathrm{OD}}_{t}}{{OD}_{0}}\right)\times 100\;$$

In order to evaluate salt tolerance, the strain was inoculated into MRS medium containing 0–10% NaCl. Following a 24 h incubation period at 37 °C, the OD_600nm_ value was measured [57]. The MRS medium was adjusted to a range of pH values from 2.0 to 11.0. The OD_600nm_ of the strain was measured after a 24 h incubation [58]. In order to assess bile salt tolerance, the strain was inoculated into MRS medium that had been supplemented with 0.3% bile salts. The OD_600nm_ was measured at 0, 2, 4, and 6 h, and the survival rate was calculated using Formula (2) [59]. Among them, OD_t_ is the optical density of the supernatant at tested times; OD_0_ is the optical density at the initial time. All experiments were performed in three biological replicates.

(2) $$\mathrm{Survival}\;\mathrm{rate}\;(\%)\;=\frac{OD_{t}}{OD_{0}}\times 100$$

### 4.5. Influencing Factors of the CFS’s Antibacterial Activity

The stability of *L. plantarum* JC2211 CFS was assessed in terms of temperature, pH and enzymatic treatments. *S.*T and *L.m* were selected as indicator strains. For thermal stability, CFS was incubated at 40, 60, 80, 100 and 121 °C for 15 min, and its antibacterial activity was then determined using the Oxford cup method [27]. For pH stability, the pH of CFS was adjusted to 2.0–7.0 [60]. In the enzymatic sensitivity assay, CFS was treated with papain, trypsin, pepsin, proteinase K and catalase (Beijing Solarbio Science & Technology Co., Ltd., Beijing, China) at a final concentration of 1 mg/mL at 37 °C for 2 h. The reaction was terminated by heating at 100 °C for 10 min. After readjusting the pH back to the initial pH 3.4, the antibacterial activity was determined [61]. Untreated CFS served as the control. All experiments were conducted in triplicate.

### 4.6. Metabolite Profiling Analysis

Metabolite profiling of CFS was conducted using LC-MS/MS. The CFS sample was lyophilized, and metabolites were extracted using methanol-acetonitrile (1:1, *v*/*v*). The mixture was centrifuged at 12,000 r/min at 4 °C. The supernatant was placed at −20 °C to precipitate proteins, followed by vacuum drying. The dried samples were redissolved in 30% acetonitrile before instrumental analysis. Chromatographic separation was performed on a Waters HSS T3 column (100 × 2.1 mm, 1.8 μm, Waters Corporation, Milford, MA, USA). Gradient elution was applied using 0.1% formic acid aqueous solution (mobile phase A) and 0.1% formic acid in acetonitrile (mobile phase B). The flow rate was 0.3 mL/min, and the column temperature was maintained at 40 °C. A Q Exactive HFX mass spectrometer (Thermo Fisher Scientific, Waltham, MA, USA) equipped with electrospray ionization (ESI) was operated in both positive and negative ion modes. The full scan was acquired over an m/z range of 70–1050 with a resolution of 70,000, while the MS/MS resolution was set to 17,500. The relative abundance of each metabolite was calculated based on its normalized peak area as a percentage of the total identified metabolite pool.

### 4.7. **Antibacterial Mechanism Assays**

#### 4.7.1. Minimum Inhibitory Concentration (MIC)

The minimum inhibitory concentration (MIC) of CFS against the four pathogenic strains was determined via the 96-well microdilution method [62]. CFS was two-fold serially diluted with LB broth, and 100 μL of each dilution was added to the wells of a 96-well plate. Next, 100 μL of bacterial suspension (~10^6^ CFU/mL) of each test strain was dispensed into the corresponding wells. The plates were incubated at 37 °C for 24 h. The MIC was defined as the lowest CFS concentration that completely suppressed visible bacterial growth.

#### 4.7.2. Membrane Permeability and Membrane Potential

Four pathogenic strains were cultured to the logarithmic growth phase (OD_600_ = 0.5) and treated with CFS at the MIC for 1 h. The treated bacterial cells were harvested by centrifugation at 8000 r/min for 5 min, washed three times with 0.85% sterile saline, and resuspended. For cell membrane permeability detection, propidium iodide (PI) was added to a final concentration of 5 μg/mL, followed by incubation at 4 °C for 30 min in the dark. The fluorescence intensity was determined using a multi-functional microplate reader with an excitation wavelength of 535 nm and an emission wavelength of 615 nm [63]. For membrane potential measurement, 3,3′-dipropylthiadicarbocyanine iodide (DiSC_3_(5)) was supplemented to a final concentration of 5 μM. After incubation at 4 °C for 1 h in the dark, the fluorescence intensity was detected at an excitation wavelength of 622 nm and an emission wavelength of 670 nm using a multi-functional microplate reader (Thermo Fisher Scientific, Waltham, MA, USA) [64]. All experiments were performed in three biological replicates.

#### 4.7.3. Intracellular ATP Content

Intracellular ATP levels were quantified using an MCE ATP Assay Kit (MedChemexpress LLC, Monmouth Junction, NJ, USA) [65]. The four pathogenic strains were cultured to the logarithmic phase and exposed to CFS at the MIC for 1 h. Bacterial cells were harvested via centrifugation at 8000 r/min for 10 min, lysed with lysis buffer, and centrifuged again at 12,000 r/min for 5 min at 4 °C. The supernatant was mixed with 100 μL working solution and incubated at room temperature for 5 min. The mixture was loaded into a 96-well plate to detect luminescence. Intracellular ATP concentration was calculated based on the standard curve (R^2^ > 0.99). All assays were carried out in three biological replicates.

#### 4.7.4. Intracellular ROS Detection

Intracellular ROS were measured using the DCFH-DA fluorescent probe (Beijing Solarbio Science & Technology Co., Ltd., Beijing, China). Bacterial suspensions were treated with CFS at the MIC for 30 min at 37 °C, then centrifuged at 10,000 r/min for 2 min. The cell pellets were rinsed three times with 0.85% sterile saline. DCFH-DA was added to the suspension to a final concentration of 2.5 μg/mL, and incubated at 37 °C for 20 min with shaking at 120 r/min in the dark. Fluorescence intensity was detected at excitation and emission wavelengths of 488 nm and 525 nm, respectively. All experiments were conducted in three biological replicates.

### 4.8. Preparation of CS/CFS Composite Films

Chitosan (CS, 2%, *w*/*v*) was dissolved in 1% glacial acetic acid solution supplemented with 0.5% glycerol, and the mixture was ultrasonicated at 45 °C for 30 min. CFS was added at volume fractions of 10%, 20%, 30%, 40% and 50%. The CS solution without CFS served as the control. All mixtures were magnetically stirred at 200 r/min for 3 h to achieve full dissolution. After defoaming, 15 mL of each solution was poured into 9 cm diameter Petri dishes and dried at 45 °C for 12 h until constant weight (mass change < 0.01 g) to form composite films [66]. Prior to any characterization or application, the dried films were conditioned at 25 °C and 50% relative humidity for 48 h to equilibrate moisture content. The films were not subjected to any sterilization treatment (e.g., autoclaving or UV irradiation) in order to preserve the bioactivity of the CFS components, as heat-based sterilization and UV irradiation could degrade heat-labile bioactive compounds and compromise the antibacterial functionality. The pure CS film served as an appropriate control to account for this background effect.

### 4.9. Antibacterial and Antioxidant Activity of CS/CFS

The antibacterial activity of the composite film was assessed via the paper disc diffusion assay [67]. Briefly, bacterial suspensions with OD_600nm_ of 0.5 were spread on LB agar plates. Paper discs were immersed in the CS/CFS film-forming solutions for 1 h and dried (45 °C for 12 h) to place on the inoculated plates. After incubation at 37 °C for 12 h, the diameters of inhibition zones were measured.

For the DPPH radical scavenging assay, 0.1 g of CS/CFS composite film was dissolved in 10 mL of deionized water, vortexed for 30 s, and then allowed to stand for 12 h at room temperature. The supernatant was collected as the test solution. Then, 1 mL of the test solution was mixed with 1 mL of 0.1 mM DPPH solution. The mixture was incubated at room temperature in the dark for 30 min, and the absorbance was recorded at 517 nm [68]. For the ABTS assay, the working solution was prepared by mixing 2.45 mM potassium persulfate with 7 mM ABTS solution at a volume ratio of 1:1 (*v*/*v*). The mixture was incubated in the dark at room temperature for 16 h and then diluted with absolute ethanol to obtain an absorbance of 0.70 ± 0.02 at 734 nm. Subsequently, 1 mL of the test solution was mixed with 1 mL of the ABTS working solution. After incubation in the dark at room temperature for 6 min, the absorbance was measured at 734 nm [69]. For both assays, a blank control was prepared by replacing the test solution with an equal volume of deionized water. The DPPH/ABTS radical scavenging activity (%) was calculated using Formula (3). $A_{0}$ is the absorbance of the blank control and $A_{1}$ is the absorbance of the sample group.

(3) $$\mathrm{DPPH}/\mathrm{ABTS}\;\mathrm{radical}\;\mathrm{scavenging}\;\mathrm{activity}\;(\%)=\frac{A_{0}\;-{\;A}_{1}}{A_{0}}\times 100$$

### 4.10. Physical Properties of CS/CFS Composite Film

Film thickness was measured using a digital micrometer with an accuracy of 0.001 mm. Five random positions on each film specimen were measured, and the average value was calculated. Tensile strength and elongation at break of the CS/CFS composite films were measured using a universal electronic testing machine. The films were cut into rectangular strips (60 mm × 20 mm) and tested at a stretching rate of 20 mm/min [70]. For microstructure observation, films were fractured in liquid nitrogen. Prior to imaging, the fractured samples were sputter-coated with a thin gold layer for conductivity enhancement. The cross-sections of the films were characterized via scanning electron microscopy (SEM, Hitachi High-Technologies Corporation, Tokyo, Japan) at magnifications of 1000× and 2500×.

### 4.11. Application of CS/CFS40 Film for Chilled Pork Preservation

Muscle tissues from the neck and back of 5–6 month-old crossbred white pigs were collected after acid maturation. After removing fascia, the pork was cut into uniform blocks, rinsed with sterile water and blotted dry with filter paper. Firstly, the artificial pathogen contamination assay was conducted. Briefly, pork blocks (5 g each) were surface-decontaminated by immersion in 150 ppm sodium hypochlorite solution for 5 min, followed by thorough rinsing with sterile water and air-drying in a biosafety cabinet [71]. The pieces were then artificially inoculated by immersion in pathogen suspensions of *S.*T and *L.m* (~10^3^ CFU/mL) for 5 min, and subsequently air-dried for 15 min. The inoculated pork pieces were divided into two groups: the treatment group was wrapped with CS/CFS40 composite film, and the control group was wrapped with pure CS film. All samples were stored at 4 °C in the dark, and sampling was performed on days 0, 1, 3, 5 and 7. For bacterial enumeration, 5.0 g of each pork sample was homogenized with 45 mL of 0.85% sterile saline, serially diluted, and spread onto selective media: *L. monocytogenes* was enumerated on PALCAM agar, and S. Typhimurium was enumerated on XLD agar. All plates were incubated at 37 °C for 24 h. All experiments were performed in three independent biological replicates, with two parallel samples analyzed per replicate per time point.

As for the chilled pork preservation, pork blocks (without disinfection) were divided into three groups: pork wrapped with CS/CFS40 composite film, pork wrapped with pure chitosan film (CS), and unwrapped pork (blank control). All samples were stored at 4 °C in the dark, and sampling was performed on days 0, 3, 6, 9 and 12 for subsequent analyses. For total viable count (TVC) determination, 5.0 g of pork was homogenized with 45 mL sterile water and shaken for 1 min. Following serial dilution, appropriate dilutions were spread onto PCA agar plates and incubated at 37 °C for 24 h to enumerate colonies [72]. The pH value was measured by inserting a portable pH meter directly into pork tissue. Color parameters (L*, a*, and b*) were detected using a colorimeter. For texture profile analysis (TPA), samples were cut into 1 cm × 1 cm × 1 cm cubes. Measurements were performed using a P36/R cylindrical stainless steel probe in TPA mode (Shanghai Bosin Industrial Development Co., Ltd., Shanghai, China) with a compression rate of 50%. The pre-test, test and post-test speeds were set to 2 mm/s, 1 mm/s and 2 mm/s, respectively. The interval between two compression cycles was 5 s, with a trigger force of 5 g. Hardness, springiness, cohesiveness and chewiness were recorded [73]. Total volatile basic nitrogen (TVB-N) was quantified using an automatic Kjeldahl analyzer in accordance with the national standard GB 5009.228-2016 [74]. For thiobarbituric acid reactive substances (TBARS) analysis, 2.5 g of minced pork was mixed with trichloroacetic acid solution and shaken vigorously. After filtration, the filtrate was blended with thiobarbituric acid solution at a volume ratio of 1:1, and the mixture was heated at 90 °C for 30 min. The absorbance was read at 532 nm, and TBARS content was calculated from the standard curve (R^2^ > 0.99) [75]. All experiments were carried out in three biological replicates; texture and color analyses were performed in five replicates.

### 4.12. Statistical Analysis

All data are presented as mean ± standard deviation. Statistical analysis was conducted using GraphPad Prism 10.1.2 and SPSS 27.0. Independent samples *t*-test and one-way analysis of variance (ANOVA) with post hoc multiple comparisons were used. A *p*-value less than 0.05 was regarded as statistically significant.

## Figures and Tables

**Table 1:** Antibiotic susceptibility of *Lactiplantibacillus plantarum* JC2211.

Strain	Antibiotic Susceptibility				
	Ampicillin (10 µg)	Chloramphenicol (30 µg)	Tetracycline (30 µg)	Gentamicin (10 µg)	Kanamycin (30 µg)
*L. Plantarum* JC2211	S	S	I	R	R
*S.a* ATCC 6538	R	R	R	R	R

Note: “S”, “I” and “R” represent antibiotic susceptibility, intermediate susceptibility, and resistance, respectively.

**Table 2:** Relative abundance of organic acids in total metabolites of *Lactiplantibacillus plantarum* JC2211.

Classification	Metabolites	Abundance (%)
Organic acids	D-Lactic Acid	9.37
	Citric Acid	8.00
	Phenyllactic Acid	4.42
	L-Leucic Acid	3.73
	2-Deoxygluconic Acid	0.68
	Succinic Acid Anhydride	0.62
	Hydroxypyruvic	0.54
	Uvic Acid	0.48
	D-Pipecolic Acid	0.41
	6-Oxopipecolinic	0.22
	D-Pidolic	0.19
	γ-Aminobutryic Acid	0.14
	Malic Acid	0.12
	2-Aminoisovaleric Acid	0.12
	Adenylic Acid	0.11
Amino acids and peptides	Amino Acid (Arg-)	1.22
	Glutaminylglutamic Acid	0.10
Benzene and aromatic compounds	Ortho-aminobenzoic acid	0.29
	Indolelactic Acid	0.21
Lipids	15-Docosenoic Acid	0.87
	Choline Alfosceric Acid	0.16
Amides	Oleic Acid Amide	0.24
	Stearic Acid Amide	0.19
	Lauric Acid Amide	0.11

Note: The relative abundance of each metabolite was calculated based on its normalized peak area as a percentage of the total identified metabolite pool.

**Table 3:** Variations in color parameters (L*, a*, b*) of pork over storage time.

Day (d)	Treatment	*L* *	a*	b*
0	Blank control	41.64 ± 2.53 ^a^	11.26 ± 0.80 ^a^	11.31 ± 0.57 ^a^
	CS	41.49 ± 2.53 ^a^	12.01 ± 0.86 ^a^	11.71 ± 0.66 ^a^
	CS/CFS40	42.06 ± 1.36 ^a^	12.06 ± 0.94 ^a^	11.49 ± 0.55 ^a^
3	Blank control	50.41 ± 2.40 ^a^	9.78 ± 1.00 ^b^	14.60 ± 1.07 ^a^
	CS	48.10 ± 1.33 ^a^	10.46 ± 0.36 ^b^	12.76 ± 0.84 ^b^
	CS/CFS40	44.05 ± 2.24 ^b^	12.16 ± 0.77 ^a^	10.91 ± 0.67 ^c^
6	Blank control	50.18 ± 1.40 ^a^	8.38 ± 0.78 ^b^	13.45 ± 1.08 ^a^
	CS	47.44 ± 2.94 ^a^	9.18 ± 0.57 ^b^	13.07 ± 0.81 ^a^
	CS/CFS40	41.87 ± 1.98 ^b^	11.36 ± 0.55 ^a^	10.53 ± 1.09 ^b^
9	Blank control	44.75 ± 2.17 ^a^	6.78 ± 0.63 ^c^	13.92 ± 1.13 ^a^
	CS	45.79 ± 2.35 ^a^	7.88 ± 0.78 ^b^	14.12 ± 0.81 ^a^
	CS/CFS40	39.93 ± 2.54 ^b^	10.13 ± 0.30 ^a^	11.64 ± 0.69 ^b^
12	Blank control	36.83 ± 2.77 ^a^	6.19 ± 0.93 ^b^	14.87 ± 0.72 ^a^
	CS	38.12 ± 2.59 ^a^	6.28 ± 0.63 ^b^	13.88 ± 0.71 ^a^
	CS/CFS40	40.15 ± 1.44 ^a^	9.64 ± 0.44 ^a^	11.13 ± 1.03 ^b^

Note: Different lowercase letters indicate significant differences among groups at the same storage time (*p* < 0.05). The unwrapped pork served as the blank control. CS represents pure chitosan film, and CFS refers to chitosan film incorporated with 40% CFS.

## Data Availability

The raw data supporting the conclusions of this article will be made available by the authors on request.

## Supplementary Materials

The following supporting information can be downloaded at: https://www.mdpi.com/article/10.3390/gels12090765/s1, Table S1 Inhibitory activity of LAB isolated from Yangzhou pickles against *Salmonella* Typhimurium (*S.*T), *Staphylococcus aureus* (*S.a*), *Listeria monocytogenes* (*L.m*), and *Pseudomonas aeruginosa* (*P.a*); Table S2 Thickness of the composite films; Figure S1 Hemolytic activity (a), proteolytic activity (b), and coagulase test results (c) of *L. plantarum* JC2211; Figure S2 Classification of identified metabolites in CFS.
