# Supplementary material for: Chitosan-Based Composite Film Containing Cell-Free Supernatant of Lactiplantibacillus plantarum JC2211 for Chilled Pork Preservation
Source: Gels. 2026 Aug 26;12(9):765. doi: 10.3390/gels12090765 (PMC13606539; doi:10.3390/gels12090765)
Supplement: Supplementary file 1 [file gels-12-00765-s001.zip › gels-4443997-supplementary.pdf]

Supplementary materials

Table S1-S2

Figure S1-S2

Table S1 Inhibitory activity of LAB isolated from Yangzhou pickles against *Salmonella* Typhimurium (*S.*T), *Staphylococcus aureus* (*S.a*), *Listeria monocytogenes* (*L.m*), and *Pseudomonas aeruginosa* (*P.a*)

Strain Number	*S.*T	*S.a*	*L.m*	*P.a*
JC1111	-	-	+	-
JC1211	+	-	-	-
JC1212	-	-	+	-
JC1214	-	+	-	-
JC1311	-	+	+	-
JC1312	-	+	+	+
JC1313	-	+	-	-
JC1314	+	-	+	-
JC1315	-	+	+	-
JC1316	+	+	+	+
JC1317	+	-	+	-
JC1411	-	-	+	-
JC1412	-	+	+	-
JC1413	-	+	+	-
JC1414	-	+	-	-
JC1415	-	+	-	-
JC1416	+	-	+	+
JC1417	-	+	+	+
JC1421	-	+	+	-
JC1422	-	-	-	-
JC1424	-	-	-	-
JC2211	+	+	+	+
JC2212	+	+	+	+
JC2213	+	+	+	+
JC2214	-	+	+	+

Note: “+” indicates the inhibitory effects, while “-” indicates the absence of inhibitory effects.

Table S2 Thickness of the composite films

Film	Thickness (mm)
Control	0.087 ± 0.009
CFS10	0.091 ± 0.020
CFS20	0.106 ± 0.038
CFS30	0.093 ± 0.026
CFS40	0.087 ± 0.017
CFS50	0.072 ± 0.011

Note: All data are expressed as mean ± standard deviation (n=5).

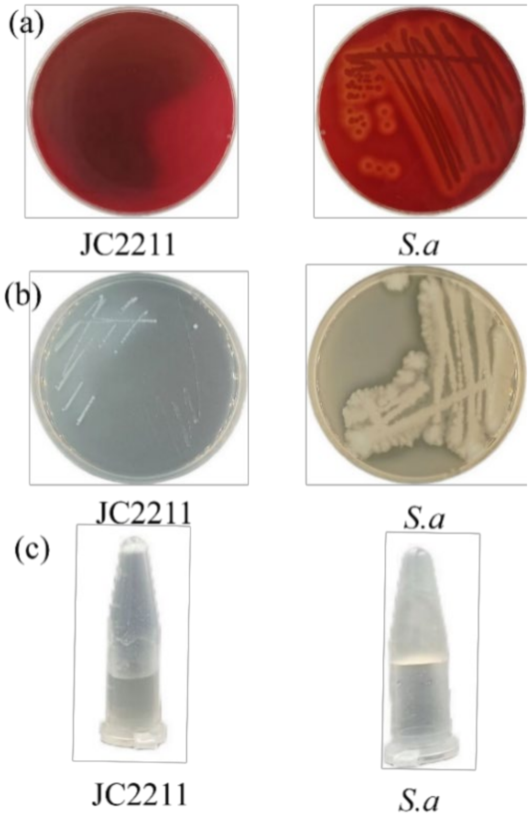

Figure S1 Hemolytic activity (a), proteolytic activity (b), and coagulase test results (c) of *L. plantarum* JC2211.

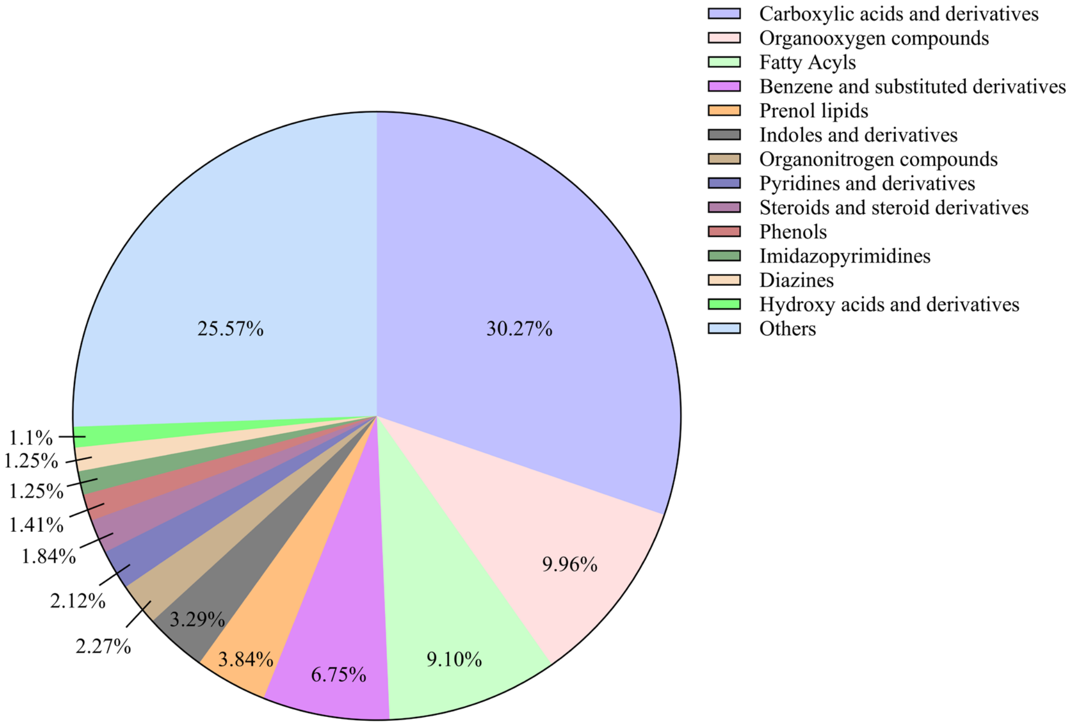

Figure S2 Classification of identified metabolites in CFS.
